# Structural and Kinetic Characterization of Thymidine Kinase from *Leishmania major*

**DOI:** 10.1371/journal.pntd.0003781

**Published:** 2015-05-15

**Authors:** Jennifer Timm, Cristina Bosch-Navarrete, Eliseo Recio, Joanne E. Nettleship, Heather Rada, Dolores González-Pacanowska, Keith S. Wilson

**Affiliations:** 1 Structural Biology Laboratory, Department of Chemistry, University of York, York, United Kingdom; 2 Instituto de Parasitología y Biomedicina López-Neyra, Consejo Superior de Investigaciones Científicas, Armilla, Granada, Spain; 3 The Oxford Protein Production Facility, Research Complex at Harwell, Rutherford Appleton Laboratory, Didcot, Oxfordshire, United Kingdom; McGill University, CANADA

## Abstract

*Leishmania spp*. is a protozoan parasite and the causative agent of leishmaniasis. Thymidine kinase (TK) catalyses the transfer of the γ-phosphate of ATP to 2’-deoxythymidine (dThd) forming thymidine monophosphate (dTMP). *L*. *major* Type II TK (*Lm*TK) has been previously shown to be important for infectivity of the parasite and therefore has potential as a drug target for anti-leishmanial therapy. In this study, we determined the enzymatic properties and the 3D structures of holo forms of the enzyme. *Lm*TK efficiently phosphorylates dThd and dUrd and has high structural homology to TKs from other species. However, it significantly differs in its kinetic properties from *Trypanosoma brucei* TK since purines are not substrates of the enzyme and dNTPs such as dUTP inhibit *Lm*TK. The enzyme had Km and k_cat_ values for dThd of 1.1 μM and 2.62 s^-1^ and exhibits cooperative binding for ATP. Additionally, we show that the anti-retroviral prodrug zidovudine (3-azido-3-deoxythymidine, AZT) and 5’-modified dUrd can be readily phosphorylated by *LmT*K. The production of recombinant enzyme at a level suitable for structural studies was achieved by the construction of C-terminal truncated versions of the enzyme and the use of a baculoviral expression system. The structures of the catalytic core of *Lm*TK in complex with dThd, the negative feedback regulator dTTP and the bi-substrate analogue AP_5_dT, were determined to 2.74, 3.00 and 2.40 Å, respectively, and provide the structural basis for exclusion of purines and dNTP inhibition. The results will aid the process of rational drug design with *Lm*TK as a potential target for anti-leishmanial drugs.

## Author Summary

The DNA within the genome of an organism encodes all the information, firstly for reproduction and secondly for translation into proteins—the workhorses of a biological cell. Proteins carry out a host of essential biological activities within the cell. A full understanding of a protein now requires determination of a wide range of its properties in solution in the cell and *in vitro* in solution, but in addition, its 3D structure usually determined by X-ray crystallography. *Leishmania* species are a family of protozoan parasites of humans and the causative agent of leishmaniasis, a major health concern in the developing world. Selective inhibition of key enzymes in these parasites is a key route for combating these diseases. We have focused our work on thymidine kinase, an important enzyme from *Leishmania major*, and a potential target for the development of new drugs. We have carried out kinetic studies of the enzyme’s activity in solution and determined its 3D crystal structure, enabling rational drug design.

## Introduction

*Leishmania* spp. are flagellate protozoan parasites of the order Kinetoplastida, transmitted by the bite of sand flies of the genera *Phlebotomus* and *Lutzomyia* [1]. They are the causative agents for a range of forms of leishmaniases, disease with approximately 1.3 million new cases and 20 000–30 000 deaths occurring annually [2]. Current treatments cause severe adverse effects, their efficiency is limited and the high costs make reliable supply in poor countries difficult, reviewed in [3]. Therefore, there is an urgent need for novel, less toxic and cost-effective anti-leishmanial compounds.

Nucleotide metabolism provides a rich source of drug targets for many diseases since a balanced pool of deoxyribonucleotides (dNTPs) is essential for DNA replication and repair and disturbances in the supply of the four dNTPs may lead to genetic mutations and eventually to cell death. In most living organisms dNTPs can be supplied by two pathways, the *de novo* and the salvage pathway [4]. *Leishmania* parasites have both pyrimidine biosynthetic *de novo* and salvage pathways but are incapable of synthesising purines *de novo* [5].

Thymidine kinases (TKs, EC 2.7.1.21) are essential enzymes for the initiation of the pyrimidine salvage pathway as they catalyse the magnesium dependent transfer of the γ-phosphate of ATP to thymidine (dThd), thus forming thymidine monophosphate (dTMP). dTMP is phosphorylated further by cellular enzymes to 2'-deoxythymidine triphosphate (dTTP), which serves as a substrate for DNA polymerase during replication [6]. Hence TKs control the dTTP pool and are important regulatory enzymes for DNA biosynthesis. Severe dTTP depletion leads to the massive incorporation of uracil into DNA and contributes to the phenomenon called “thymineless death” [4]. TKs are classified into two subgroups (types I and II) according to their sequence and substrate specificity. Type I TKs include herpes simplex virus TK (HSV-TK) and mitochondrial human TK2. The much more widely spread type II TKs are present in most organisms: eukaryotes (for example protozoan parasites like *Leishmania* spp. and *Cryptosporidium* spp.; plants; and cytosolic human TK1), bacteria (including *Escherichia coli*, *Ureaplasma urealyticum*, *Bacillus anthracis*, *Mycoplasma pneumoniae*) and many viruses such as variola, vaccinia and poxviridae [7–14].

Human TK1 (*h*TK1) is the most studied type II enzyme highlighting key features of this subfamily of kinases. Its expression is tightly regulated throughout the cell cycle due to specific S-phase regulated expression, reaching maximum levels in proliferating tissues, and increased TK activity in cancer cells can be used as a tumour marker [15–17]. Alterations in TK knock out mice indicate that the pyrimidine nucleotide salvage pathway is indispensable *in vivo* [18]. Type II TKs are highly specific towards nucleotide substrate accepting only thymidine or uracil as base, or analogues with minor changes in the 5-position of the pyrimidine base and/or in the 3’-position of 2’-deoxyribose sugars [19].

The enzymes from *Leishmania* spp. are type II TKs with a ~100 amino acid C-terminal extension compared to those from other species. The function of this extension is not known. *Leishmania donovani* TK (*Ld*TK) has been shown to be located in the cytosol. *L*. *major* TK (*Lm*TK) knockout mutants show morphological defects, lower proliferation rates and are less infective [13]. Since TK activity is important for *Leishmania* spp., the aim of the present study was the purification and structural and kinetic characterization of recombinant type II *Lm*TK to identify differences from the human enzyme which might be exploited in drug design.

## Materials and Methods

### Bacterial strains, plasmids and chemical reagents

The pET28a and pET22b expression system, *E*. *coli* BL21(DE3), XL-1Blue strains and benzonase were purchased from Novagen. The pGEMT vector, Wizard *Plus* SV Minipreps DNA Purification System and FuGene® HD transfection reagent were from Promega. The In-Fusion™ enzyme was purchased from Clontech-Takara Bio Europe. L-lactate dehydrogenase from rabbit muscle, restriction enzymes and protease inhibitor cocktail were supplied by Roche. All nucleosides and nucleotides were purchased from Sigma-Aldrich and Jena Bioscience.

### Cloning of the *Lm*TK gene for expression in *E*. *coli*

The *Lm*TK gene (GeneDB LmjF.21.1210) was amplified using the PCR Extender System, from genomic DNA of *L*. *major* strain Friedlin with oligonucleotide primers 5´-**CAT ATG** TTC CGC GGT CGT ATA GAG-3´ and 5´-**CTC GAG** TCA CTC TGA GGA TGC AGC-3´ that include NdeI and XhoI restriction sites (in bold), respectively. The amplified full-length *Lm*TK was cloned into the pGEMT vector and subsequently ligated into the NdeI and XhoI sites of pET28a vector. The resulting construct contained a His-tag at the N-terminus. Additionally *Lm*TK was cloned into the expression vector pET22b, which carries a C-terminal His-tag sequence using the restriction sites of *Nde*I and *Hind*III. Plasmid DNA was isolated with the Wizard *Plus* SV Minipreps DNA Purification System (Promega). Positive clones were verified by genomic digestion and double-stranded DNA sequencing.

### Protein expression in *E*. *coli* and purification

Recombinant full-length *Lm*TK was overexpressed from the pET28-*Lm*TK vector in *E*. *coli* BL21 (DE3) and grown in LB medium supplemented with kanamycin 50 μg/ml. Protein expression was induced with 0.5 mM IPTG for 5–6 hours and purification performed using a 1 ml HisTrap column. Cell pellets obtained by centrifugation of a 2 L induced culture were washed with PBS, pH 7.2. The pellet was resuspended in buffer A (20 mM Tris-HCl, 10 mM MgCl_2_, 0.5 M NaCl, 1 mM PMSF and 20 mM imidazole, at pH 7.4) supplemented with a protease inhibitor cocktail. Bacterial cells were then lysed by discontinuous sonication. The cell extract was cleared by centrifugation at 11 000 x *g* for 30 min at 4°C and the supernatant loaded onto an equilibrated 1 mL HisTrap column connected to an AKTA purifier (GE Healthcare). The column was washed with 10 mL of lysis buffer and the protein eluted using an imidazole gradient from 20 mM to 1 M, at a flow rate of 0.5 ml/min.

Fractions containing *Lm*TK were pooled and desalted using a PD-10 column. The enzyme was eluted with 20 mM Tris-HCl pH 8.0, 10 mM MgCl_2_, 0.15 M NaCl and concentrated by centrifugation in 10 KDa Centriprep centrifugal devices. The yield of purified protein was 4–6 mg/L. Purified *Lm*TK was stored at -80°C after addition of 1 mM DTT and 50% (v/v) glycerol. Protein identification by peptide mapping fingerprinting and molecular mass determination were performed at the Proteomics Unit of the Institute of Parasitology and Biomedicine López-Neyra using a Voyager DE-PRO MALDI-TOF spectrometer (Applied Biosystems, USA). This material was sufficient for kinetic analyses.

For structural studies, a total of 26 full-length and truncated *Lm*TK constructs were tested for expression using various *E*. *coli* strains, different media, induction systems and temperatures. Additionally more than 30 different cell lysis conditions and various buffer systems were tried for stabilisation of the protein. However, none gave sufficient amounts of soluble and stable protein for crystallization screening as it lost enzymatic activity and precipitated within 24 h at room temperature and 4°C. Therefore, expression in insect cells using the baculovirus system was used, see below.

### Activity assay

TK activity was measured spectrophotometrically using an enzyme coupled assay [20] in buffer containing 50 mM Tris-HCl pH 7.4, 5 mM MgCl_2_, 50 mM KCl, 1 mM DTT, 0.5 mM ATP, 0.5 mM phosphoenolpyruvate (PEP), 0.2 mM NADH, 0.2 mM thymidine (dThd), 2 U pyruvate kinase (PK), 4 U lactate dehydrogenase (LDH) and variable amounts of enzyme. The reaction rate (AU/min) was registered as a decrease in absorbance at 340nm (NADH extinction coefficient) at 25°C at pH 7.4 using a multicell Hewlett-Packard 8452 diode array UV-VIS spectrophotometer. Diluted stocks of frozen full-length *Lm*TK were freshly prepared in assay buffer before the measurements.

### Nucleotide analysis by HPLC

Complementary activity measurements were carried out using a direct method based on the HPLC separation of nucleotide substrates and products and their UV-visible detection at 254 nm using an AKTA purifier [21]. The number of phosphate groups determines their different times in a MonoQ 5/50 GL column (GE Healthcare). The reaction was performed in 100 μl of buffer containing 50 mM Tris-HCl pH 7.2, 40 mM KCl, 2 mM MgCl_2_, 0.5 mM DTT with 11 μg of enzyme and 50 nmol of dThd and ATP. The reaction mixture was incubated for 10 minutes at 25°C before being loaded onto the column. Reaction products were eluted in NaH_2_PO_4_ pH 7, at a flow rate of 0.3 ml·min^-1^ with a salt gradient of 50 to 350 mM NaCl. Standard solutions of dThd, ATP, ADP and dTMP were injected and eluted under the same conditions to determine their retention times.

### Inhibition assay

The method consists in determining experimental values for the initial velocities, v, as described above, at increasing inhibitor concentrations, added before initiating the reaction. *Lm*TK and dThd concentrations were 22.3 nM and 25 μM respectively. The concentration of inhibitor that inhibits the activity by 50% (IC_50_) was calculated.

### Construction of baculoviruses

Recombinant full length and a C-terminal truncated version of *Lm*TK spanning amino acids 2–185, were expressed in insect cells using the baculovirus system. Transfer vectors were constructed by ligation-independent cloning [22] using In-Fusion™ enzyme (Clontech-Takara Bio Europe) and incorporated either a N-terminal His_6_ tag, cleavable by rhinovirus 3C protease, or a C-terminal His_6_ tag. Baculoviruses were constructed as follows. *Sf9* cells were grown in Sf900 II-SFM media and plated in 24-well plates to a density of ~ 5 x 10^5^ cells·mL^-1^ at 500 μL per well. Attachment to the well surface was allowed in 1 h incubation at room temperature prior to co-transfection. For transfection 4 μL (100 -500 ng depending on construct) of pOPIN transfer vector, 2.5 μL (500 ng) linearised *Autographa californica* bacmid [23], 0.75 μL FuGene® HD (Promega) and 50 μL Sf900 II-SFM were mixed and pre-incubated for 30 min at room temperature before addition to the adherent *Sf*9 cells. The primary viral stocks were harvested manually from the media 168 h post-transfection. These virus stocks were used for infection of fresh *Sf*9 cells, which were grown to a density of ~1 x 10^6^ cells·mL^-1^, plated in 24-well plates with 500 μL per well and allowed to attach to the well surface 1 h prior to the infection. Adherent cells were infected with 5 μL primary virus stock. Final virus stocks were harvested manually from the supernatant 168 h post-infection and stored at -80°C.

### Protein expression in Sf9 insect cells and purification

A small-scale expression test was set up using 5 mL *Sf*9 cultures and two viral concentrations (5 μL or 50 μL of virus per 5 mL of culture) and two harvest points (48 and 72 hours). Soluble expression was determined using a small-scale Ni-NTA purification followed by SDS-PAGE analysis [22]. The optimal virus volume/cell and time of harvest determined in the small-scale expression screen were used for scaling up protein expression. Thus 1 L cultures at 1 x 10^6^ cells·mL^-1^ were infected with 500 μl of amplified virus and incubated at 26°C for 72 hours. Cells were harvested by centrifugation for 30 min at 5000 x *g*.

Cell pellets from each 1 L culture were resuspended in 35 mL lysis buffer (20 mM Tris pH 8.0, 300 mM NaCl, 30 mM imidazole, 1 mM DTT and 100 μM PMSF) and lysed by sonication. This was followed by addition of 25–29 U benzonase to degrade nucleic acids, incubating for 10 min at room temperature. The cell debris was removed by centrifugation for 30 min at 5000 x *g* and 4°C and the supernatant loaded onto an equilibrated 1 mL HisTrap crude FF column for 1 h in a loop on ice. After washing with 10 mL lysis buffer the column was connected to an AKTA purifier, washed with another 10 mL of lysis buffer and eluted with an imidazole gradient from 30 mM to 500 mM. The protein containing fractions were pooled and concentrated up to 4 mL total volume. In order to stabilise the protein, it was diluted with 1 mL Size Exclusion Chromatography (SEC) buffer (10 mM Tris pH 8.0, 200 mM NaCl, 1 mM DTT) containing 1 μM ZnSO_4_.

The construct with a non-cleavable C-terminal His-tag was concentrated further to 250 μL total volume. Size exclusion chromatography on an equilibrated Superdex 200 column was carried out in SEC buffer (as above). Protein-containing fractions were pooled and in order to prevent protein precipitation and degradation, it proved necessary to stabilise the solution by addition of either 1 mM dThd, 1 mM AppNHp and 3 mM MgCl_2_ or 1 mM dTTP and 3 mM MgCl_2_. The first condition was aimed at crystallising a complex with dThd and AppNHp, the second at a complex with dTTP. The stabilised protein was concentrated to 2.4 mg·mL^-1^ (dThd, AppNHp, MgCl_2_) and 7.3 mg·mL^-1^ (dTTP, MgCl_2_), aliquoted, flash frozen in liquid nitrogen and stored at -80°C.

*Lm*TK with a cleavable N-terminal His-tag was diluted with SEC 1:2. To remove the His-tag HRV 3C protease was added in a ratio of 1:100 (protease:*Lm*TK) and incubated at 4°C overnight. Proteolysis was monitored by SDS-PAGE and reached ~90% after 16 h. The protein buffer was exchanged into lysis buffer using Centrifuge filters (Amicon® Ultra Centrifugal Filter from Millipore, 30 kDa cutoff) and nickel affinity purification performed as above. The tag-free *Lm*TK in the flowthrough was concentrated to 250 μL total volume and injected onto an equilibrated Superdex 200 column and the final purity analysed by SDS-PAGE. Protein-containing fractions were pooled and concentrated to 15.1 mg·mL^-1^ in the presence of 1 mM dThd,1 mM AppNHp and 3 mM MgCl_2_. Concentrated protein was aliquoted, flash frozen in liquid nitrogen and stored at -80°C.

Prior to crystallization the polydispersity was determined by dynamic light scattering (DLS) at 20°Cusing a DynaPro™ (Protein Solutions™). The protein was diluted in SEC buffer to a total concentration of 3 mg·mL^-1^ with a total sample volume of 20 μL.

### Analytical ultracentrifugation of *Lm*TK

C-terminal histagged *Lm*TK stabilized with dTTP was dialyzed against SEC buffer to remove dTTP and subsequently diluted with dialysis buffer to 0.15 mg·mL^-1^ and 0.45 mg·mL^-1^. C-terminal histagged *Lm*TK stabilized with dThd and AppNHp was dialyzed against SEC buffer containing 1 mM dThd and 1 mM AppNHp and diluted in dialysis buffer to 0.15 mg·mL^-1^ and 1.0 mg·mL^-1^. Analytical ultracentrifugation on all samples was carried out with a Beckman Optima XL/1 (Beckman Coulter) using an AN50Ti rotor at 42 000 rpm and 20°C. Sedimentation was recorded using the refractive index with the respective dialysis buffers as reference.

### Crystallization, data collection and structure solution

Commercial screens (Index, PACT and JCSG) were set up in sitting-drop vapour diffusion format with 150 nL protein solution including additives plus an equal volume of precipitant using a Mosquito robot (TTP Labtech, UK). Crystals were optimised with 1 μL protein solution plus an equal volume of precipitant. The crystal used to solve the structure in complex with dThd and phosphate (*Lm*TK-dThd), was grown in 1.4 M Na/K PO_4_ pH 5.4, 1 mM dThd, 1 mM AppNHp and 3 mM MgCl_2_ and had a hexagonal rod shape with approximate dimensions 300x100x100 μm^3^. The crystal of the *Lm*TK-dTTP complex was grown from 50 mM HEPES pH 8.0 and 36% (w/v) penta-erythritol-propoxylate with 1 mM dTTP and 3 mM MgCl_2_ added to the protein, and had an irregular shape reaching a maximum size of ~30 x 30 x 60 μm^3^. The crystal leading to the complex with P1-(5-adenosyl)-P5-(5-thymidyl) pentaphosphate (*Lm*TK-AP_5_dT) showed a needle-like morphology of ~50 μm length and grew in 0.1 M tri-sodium citrate pH 5.5 and 20% (w/v) PEG 3000 with addition of 1 mM AP_5_dT to protein already containing 1 mM dThd, 1 mM AppNHp and 3 mM MgCl_2_. The crystals were not cryo-protected before vitrification.

Diffraction data of all three crystals were collected at the Diamond Light Source (DLS). All computations were carried out using the CCP4 software package [24]. The images were processed with MOSFLM [25] and AIMLESS [26]. Details of data collection, crystallographic data and statistics are summarised in Table 1.

**Table 1:** Crystallographic data and statistics.

	*Lm*TK-dThd-AppNHp	*Lm*TK-dTTP	*Lm*TK-AP_5_dT
**Data collection**			
Diffraction Source	DLS beamline I04	DLS beamline I04	DLS beamline I24
Wavelength (Å)	0.9795	0.9795	0.9686
Temperature (K)	100	100	100
Detector	Pilatus 6M-F	Pilatus 6M-F	Pilatus3 6M
Rotation range per image (°)	0.2	0.2	0.1
Total Rotation range (°)	72	80	90
**Crystal data**			
Space group	P6_5_22	P1	P6_5_22
a, b, c (Å)	121.1, 121.1, 116.2	61.60, 61.63, 110.1	64.15, 64.15, 324.6
α, β, γ (°)	90, 90, 120	81.07, 85.8, 74.94	90, 90, 120
Resolution (Å)	52.46–2.74 (2.88–2.74)	59.45–3.00 (3.16–3.00)	45.84–2.40 (2.49–2.40)
Total Reflections	271650 (35991)	58503 (8708)	137341 (12676)
Unique Reflections	13223 (1772)	29290 (4271)	16728 (1684)
Completeness (%)	96.3 (100)	94.7 (94.7)	99.9 (99.6)
Redundancy	20.5 (20.3)	2.0 (2.0)	8.2 (7.5)
R_merge_	0.110 (1.174)	0.135 (0.600)	0.118 (0.690)
Mean [I/σ (I)]	17.3 (2.9)	6.2 (2.1)	11.0 (2.7)
Matthews’ coefficient (Å^3^/Da)	2.93	2.26	2.19
Protomers per AU	2	8	2
**Refinement statistics**			
Reflections, working set	12506	27884	15762
Reflections, test set	648	1404	829
Resolution Range (Å)	52.46–2.74	59.45–3.00	45.84–2.40
R-factor	0.2822	0.2603	0.1955
R_free_	0.3104	0.2784	0.2569
**No. of non-H atoms**			
Protein	2370	9673	2598
Ligand	49	232	112
Water	13	53	57
**Mean B factors (Å** ^**2**^ **)**			
Protein	88.8	49.5	29.8
Ligands	71.7	38.1	51.6
Water	57.1	22.8	40.4
**RMS deviation from ideal**			
Bond length (Å)	0.0065	0.0094	0.0124
Bond angles (°)	1.2916	1.4449	1.6943
**Ramachandran Plot** ^**1**^ **(%)**			
Residues in favored region	94.94	96.00	95.43
Residues in allowed region	5.06	4.00	4.57
Outliers	0	0	0
Molprobity score	1.67	1.69	1.07
Poor Rotamers^2^ (%)	0	0.11	0.75
**PDB ID**	**4uxi**	**4uxj**	**4uxh**

^1^ Ramachandran Plot generated with COOT (Emsley *et al*., 2010).

^2^ Poor Rotamer analysis performed using *Molprobity* (Chen *et al*., 2010).

The structures were solved by molecular replacement using PHASER [27] using the structure of *T*. *brucei* TK (*Tb*TK) (manuscript in preparation) with 58% sequence identity as search model. The *Lm*TK-dThd and *Lm*TK-AP_5_dT structures had two protomers (half a tetramer) in the asymmetric unit, whilst the *Lm*TK-dTTP complex had two tetramers in the asymmetric unit. Refinement was performed using REFMAC5 [28] with one TLS group per chain and local NCS restraints automatically generated by REFMAC5. For the first few rounds external restraints generated from *Tb*TK using ProSMART [29] were applied. The last rounds of refinement were carried out with the jelly body option with sigma 0.02. Model building and fitting of the ligands were carried out in COOT [30] and the models were validated in COOT and *MolProbity* [31].

## Results and Discussion

### Expression and purification

Full-length *Lm*TK was expressed in *E*. *coli* with N-terminal and C-terminal His-tag fusions. Both were catalytically active and the N-terminal His-tagged *Lm*TK was successfully purified using metal affinity chromatography. The enzyme was stable at—80°C for several weeks and enzyme purity was assessed by SDS-PAGE that gave a single band with minor contaminants that accounted for less than 3% of the total protein. The identity of the protein was confirmed by tryptic digestion and peptide fingerprinting and the minor contaminants were identified as mostly degradation products of *Lm*TK. The molecular mass of the major component was 33602 ± 5 Da in agreement with that calculated for the His-tagged *Lm*TK missing the initial methionine residue (33733.5 Da), with a second significant peak at 33781 Da possibly caused by an oxidized cysteine residue. Kinetic measurements and characterization of the enzyme were carried out using this preparation.

Although the yield and stability of full-length *Lm*TK produced in *E*. *coli* was adequate for kinetic measurements, it was not sufficient for structural studies. Therefore, protein expression was switched to insect cells using the baculovirus system and a C-terminally truncated *Lm*TK, spanning amino acids 2–185, was successfully produced for crystallization trials. The truncated version was designed based on homology to other TKs (alignment in S1 Fig) to exclude disordered regions (S2 Fig) with a very high theoretical pI predicted using both RONN [32] and XtalPred-RF [33,34]. Versions with either a cleavable N-terminal His tag or a C-terminal His tag were constructed and both gave similar yields of protein (~ 3 mg per L culture). The His-tag was cleaved from the N-His construct by digestion with 3 C proteases to yield untagged *Lm*TK. To prevent precipitation during purification either dThd and AppNHp or dTTP were added. Protein purity was monitored by SDS-PAGE and no degradation could be detected. DLS analysis showed the C-terminal His-tagged truncated *Lm*TK to be monodisperse while the untagged protein displayed a polydispersity of 45.3%. Despite the polydispersity of the latter sample, both protein preparations were successfully used for crystallization screening.

### Substrate specificity

The specific activity under the standard assay conditions at saturating concentrations of dThd and ATP was 4.5 μmol·min^-1^·mg^-1^, similar to that reported for *T*. *brucei* TK (*Tb*TK) [35]. The activity was examined in the pH range 5–10 and showed an optimum at pH 8.0 (S3 Fig), thus within the range for maximum cytosolic *h*TK1 activity [36,37]. The nature of the reaction products was confirmed using HPLC.

**Fig 1:**
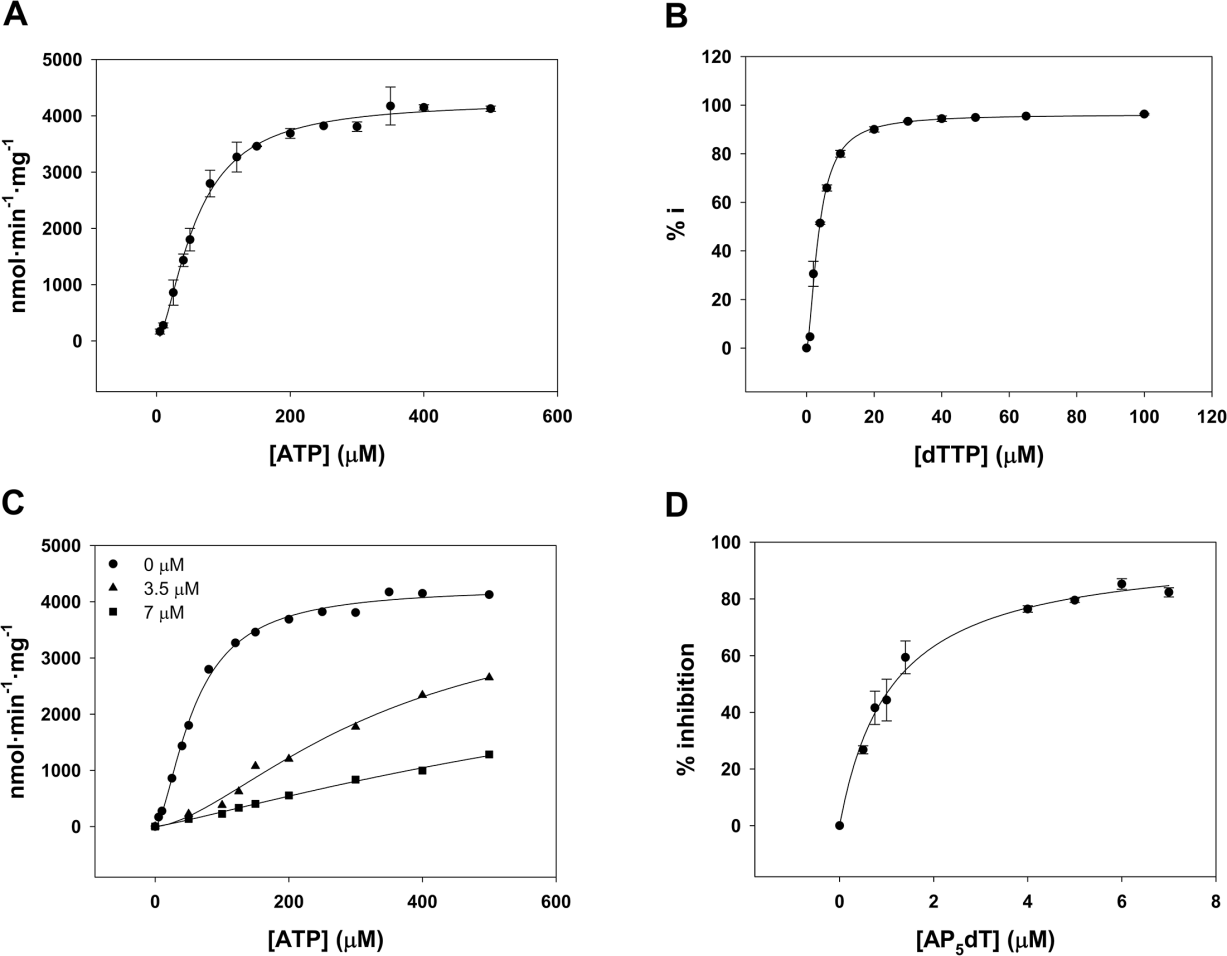
Kinetic analysis of *Lm*TK. (A) ATP substrate dependent specific activity. Experiments were performed at 25 μM dThd and kinetic constants obtained by a least-squares best fit of the experimental data to the Hill equation. (B) Inhibition of enzyme activity by dTTP. Experiments were performed at 25 μM dThd and 500 μM ATP. The IC_50_ value was calculated by non-linear regression analysis of the data. (C) The modulating effect of ATP on the inhibition of *Lm*TK by dTTP. dTMP formation was monitored at variable ATP concentrations and 25 μM dThd; in the absence of dTTP and in the presence of 3.5 μM and 7 μM dTTP. (D) Inhibition of enzyme activity by AP_5_dT. Experiments were performed at 25 μM dThd and 500 μM ATP. The IC_50_ value was calculated by non-linear regression analysis. Each point represents the average of three determinations, and error bars represent the standard deviation.

*Lm*TK displays positive cooperativity for ATP (Hill coefficient 1.6) with a K_0.5_ of 58.6 ± 2.4 μM at saturating dThd concentrations (Fig 1A). This behaviour is consistent with the reported modulation of some TK1-like enzyme activities upon ATP binding, mediated by the catalytic residues of the P-loop, which are highly conserved [36,38–42]. *Lm*TK efficiently phosphorylates its natural substrates (dThd and dUrd), with the K_M_ for dU being approximately 10-fold higher than that for dThd (Table 2). The K_M_ for dThd was 1.1 μM, ~ 7-fold lower than that reported for *Tb*TK [35]. The recombinant enzyme phosphorylates Urd but to a much lower extent (K_M_ = 1462 μM). In contrast, purines and pyrimidines are only very poor substrates. Thus, at 10 mM, the rate of phosphorylation of dIno, dGuo and dAdo, by *Lm*TKwas 23, 10 and 6 fold lower respectively than the Vmax value exhibited by *Tb*TK for these substrates. Kinetic parameters for the substrates dUrd, 5F-2’dU and AZT were similar to those previously reported for the human enzyme (Table 2, [43]).

**Table 2:** Kinetic constants and substrate specificity of recombinant *Lm*TK and *h*TK1.

Substrate	K_M_	V_max_	k_cat_	k_cat_/K_M_
	(μM)	(μmol∙min^-1^∙mg^-1^)	(s^-1^)	(s^-1^.M^-1^)
***Lm*TK**				
dThd	1.10 ± 0.33	4.66 ± 0.63	2.62	2.38 x 10^6^
dUrd	11.1 ± 2.7	5.45 ± 0.77	3.06	2.76 x 10^5^
dCyd	N.D.	0.07^a^		N.D.
dAdo	N.D.	0.01^a^		N.D.
dGuo	N.D.	0.04^a^		N.D.
Urd	1462 ± 158	0.79 ± 0.12	0.44	3.03 x 10^2^
Cyd	N.D.	0.00^a^		N.D.
Ado	N.D.	0.00^a^		N.D.
Guo	N.D.	0.00^a^		N.D.
dIno	N.D.	0.04^a^		N.D.
AZT	3.04 ± 0.76	3.52 ± 0.56	1.97	6.51 x 10^5^
5F-2’dU	5.72 ± 0.56	8.29 ± 1.12	4.66	8.14 x 10^5^
5Br-2’dU	2.05 ± 0.33	6.17 ± 0.19	3.47	1.69 x 10^6^
5CH_2_OH-2’dU	96.7 ± 23.1	5.01 ± 1.30	2.82	2.91 x 10^4^
5F-5’dU	N.D.	0.00^a^		N.D.
***h*TK1** ^**b**^				
dUrd	9.0	11.87	5.07	0.56 x 10^6^
AZT	0.6	4.96	2.12	3.53 x 10^6^
5F-2’dU	2.2	8.99	3.84	1.74 x 10^6^

Kinetic parameters for *Lm*TK are means of two or more independent experiments.

N.D. Not determined.

^a^ Activity measurements at 10 mM substrate concentration.

^b^ Kinetic constants obtained at 37°C by Munch-Peterson et al. [43].

In addition, various sugar and base modified dUrd and dThd analogues were tested (Table 2). Deoxyuridine analogues with halogens at position 5 were good substrates exhibiting K_M_ for 5F-2’dU and 5Br-2’dU of 5.7 and 2.0 μM respectively and k_cat_/K_M_ values higher than those for dU. 5CH_2_OH-2’dU is also phosphorylated while 5F-5’dU is not. The enzyme can also activate ziduvudine (3’-azido-3’-deoxythymidine, AZT), with a K_M_ of 3.0 μM.

### Enzyme inhibition

TK activity is strictly regulated to maintain the nucleotide pool. The end product of the thymidine salvage pathway, dTTP, exerts strong feedback inhibition, limiting the phosphorylation of lethal nucleoside analogues or DNA chain terminators both in mam006Dalian and in *T*. *brucei* TKs [35,44–48]. The inhibition by dTTP was first measured with *Lm*TK at saturating ATP levels (0.5 mM) and equimolar concentrations of dTTP and dThd (0.2 mM) resulting in 98% enzyme inhibition. Comparable results were obtained for partially purified TK from rat liver (87% inhibition) under similar conditions [44]. The determination of the IC_50_ value for dTTP was performed at increasing dTTP concentrations using saturating concentrations of ATP (0.5 mM) and dThd (25 μM) and gave a value of 3.8 ± 0.07 μM and a positive Hill coefficient (h = 1.6) (Fig 1B). On the other hand, an analysis of the inhibition of 3.5 μM dTTP at variable ATP concentrations (50–500 μM) and 25 μM dThd showed a 5-fold increase in the K_0.5_ value for ATP compared to that in the absence of dTTP (Fig 1C). It is important to emphasize that all dTTP experiments were performed at pH 7.4 as it has been reported that small fluctuations around this value modulate the feedback inhibition since dTTP has a pKa_2_ of 7.5 [48].

Potential inhibition by other nucleotides was monitored. It has previously been described that other dNTPs have no effect on *Tb*TK activity [35,44]. Here we found that dUTP and to a much lower extent dGTP and dCTP, inhibit *Lm*TK with IC_50_ values of 187 μM, 549 μM and 1252 μM respectively. dTMP also inhibits the activity with an IC_50_ of 165 μM. The purine nucleoside adenosine shows no inhibition when tested at 5 mM.

Bisubstrate analogues, with thymidine and adenosine linked by a phosphoryl chain (Ap_n_dT), have been identified as potent inhibitors of *h*TK1 and TMPK. Ap_5_dT proved to be the most active with a K_i_ of 0.12 μM for both enzymes [49]. Ap_5_dT was also a strong inhibitor of *Lm*TK with an IC_50_ value of 1.1 μM (Fig 1D).

### Crystal structures

*Lm*TK significantly differs in its primary sequence from *Tb*TK since the latter is a tandem protein composed of two TK domains [35] while the *L*. *major* enzyme contains a single TK domain. The *Lm*TK constructs forming diffracting crystals were heavily truncated, lacking the largely disordered C-terminal extension of unknown function. No screens were performed with the apo-enzyme as it was necessary to add ligands to achieve stable and soluble protein. *Lm*TK was crystallised with the substrate dThd plus the non-hydrolysable ATP-analogue AppNHp as the phosphate donor, and in the presence of the inhibitors dTTP and AP_5_dT. Seeding experiments failed to improve the crystal quality for the dThd-AppNHp or dTTP crystals.

**Fig 2:**
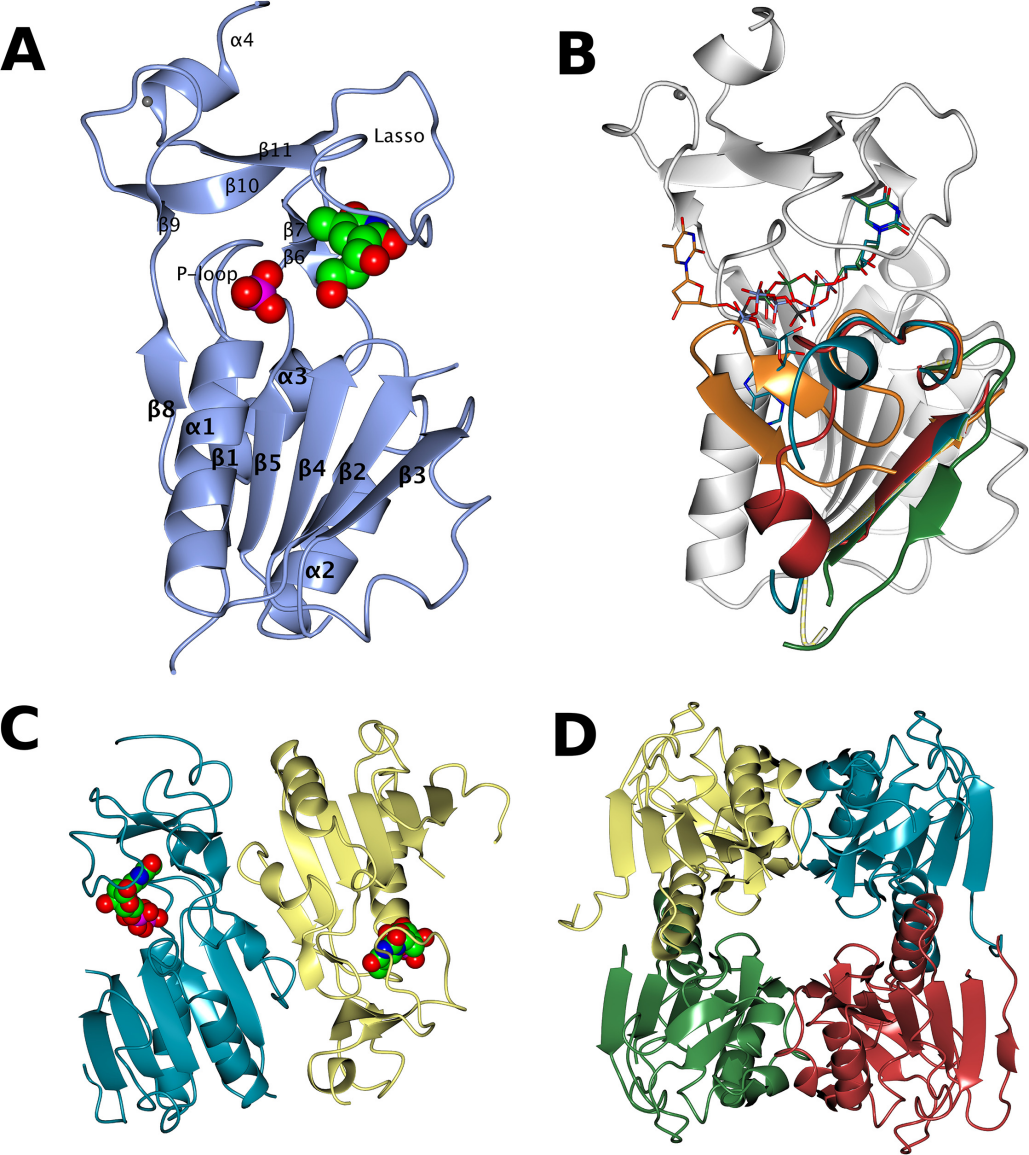
Structure of *Lm*TK. (A) The protomer in complex with dThd and phosphate, ligands as spheres coloured by atom type. (B) Superposition using SSM [55] showing the loop region corresponding to *Lm*TK residues 42–59 of *Lm*TK-dThd in lemon, *Lm*TK-dTTP in green, *Lm*TK-AP_5_dT in ice blue, *Bc*TK (2ja1) in orange and hTK1 (1w4r) in pale crimson. The overall fold of *Lm*TK-dThd is shown in white. (C) The dimer in the asymmetric unit in the dThd-phosphate complex with the ligands shown as spheres coloured by atom type. (D) The tetramer generated using PISA [56]. Figure made using CCP4mg [57].

In all three structures analysis with PISA [50] suggest the tetramer as the most likely biological entity, in keeping with all structures of type II TKs in the PDB. The overall structure of the *Lm*TK catalytic domain in all three complexes shows a nearly identical fold to those of type II TKs from other species, as expected from the sequence identity of ~50% on the amino acid level. The structure superposes with *h*TK1 (PDB: 1w4r, [51] [Welin, 2004 #14] with an overall RMSD of 0.32 Å. Each chain shows the typical two-domain structure with the active site sitting between the domains (Fig 2A, [51] [Welin, 2004 #14]. In brief, the big N-terminal α/β-domain consists of a central six-stranded parallel β-sheet (β1, β2, β3, β4, β5, β8), flanked by a long α-helix (α1) and a loop region of 15–20 amino acids on one side and two shorter α-helices (α2 and α3) and a loop on the other. The conserved P-loop, responsible for phosphate binding and essential for the kinase activity, is located between β1 and α1.

The loop region corresponding to residues 42–59 in *Lm*TK is disordered in most TK structures in the PDB, only taking up an ordered conformation in a few chains of *Uu*TK (2b8t, [52], *Bc*TK (2ja1, [53], hTK1 (1w4r, [36] and *Ca*TK (1xx6) (Fig 2B). In most chains of the *Lm*TK structures this loop is also disordered. While in *Lm*TK-dTTP it can be traced in two out of the eight chains, this is an artefact of crystallization as it forms an additional β-strand to the central β-sheet of a symmetry-related tetramer in the crystal lattice. Additionally, chain A of *Lm*TK-AP_5_dT shows a partial folding of this loop over the adenosine moiety, but the adenosine is bound in a different position to that expected from comparison with other TK structures (*Ca*TK-ADP: 1xx6, *Tm*TK-dThd-AppNHp: 2qq0, *Tm*TK-Ap_4_dT: 2orw, *Bc*TK-dTTP: 2ja1).

The smaller C-terminal domain contains three β-strands (β9–11) and a short α-helix (α4) at the C-terminus. A structural Zn^2+^ ion is tetrahedrally coordinated by four cysteines, C140 and C143 in the loop between β9 and β10 and C173 and C176 at the start of α4. The long loop between β10 and β11 forms a lasso structure covering the thymidine binding site, held in place by residues R153 and Y169, stabilising the conformation of the main chain through hydrogen bonds. Y169 is conserved in all TK sequences, R153 is changed to lysine in the *Thermotoga maritima* enzyme.

### Substrate and inhibitor binding

A number of published TK structures had revealed the binding sites for thymidine in a deeply buried pocket [51,53]. In contrast there was no ordered adenine in most of the TK complexes prepared with the aim of identifying the phosphate donor (usually ATP) site, with two exceptions. The structures of *Ca*TK with ADP (1xx6) and *Tm*TK with dThd and AppNHp (2qq0) and Ap_4_dT (2orw) had adenosine bound in an equivalent position with the ATP in an exposed site on the protein surface. In addition, there are structures of complexes of bi-substrate mimics with extended phosphate linkers, but in these the presence of the additional phosphate groups clearly mean that the polyphosphate chain must follow a different path from that in the real substrate/transition state.

**Fig 3:**
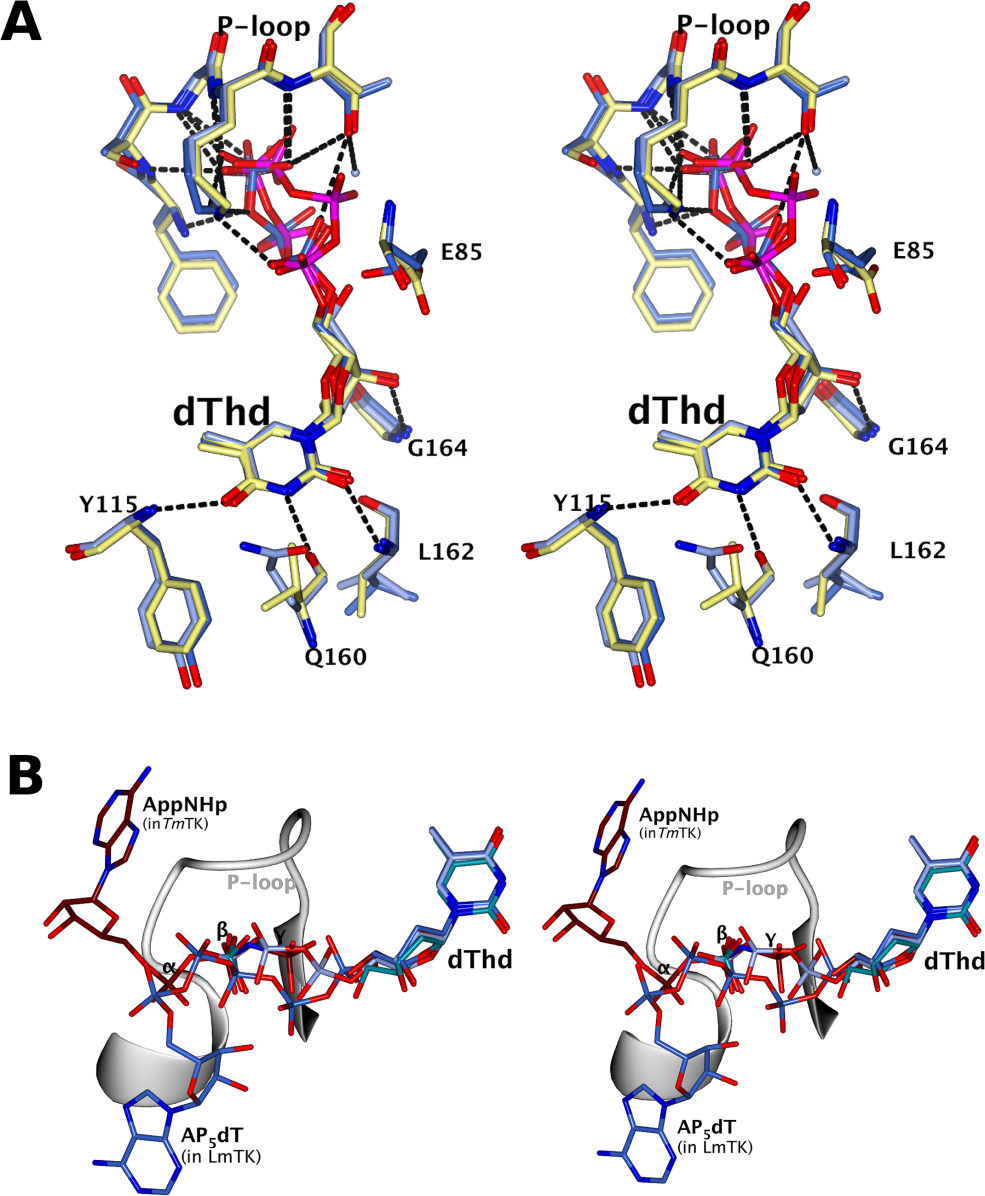
Stereo views of the ligand sites after superposition with SSM [55] in CCP4mg [57]. (A) The dThd binding site of *h*TK1 (lemon), *Lm*TK-dThd (light blue) and *Lm*TK-dTTP (ice blue). (B) *Lm*TK-dThd (dark cyan), *Lm*TK-dTTP (ice blue), *Lm*TK-AP_5_dT (light blue) and *Tm*TK-dThd-AppNHp (tan). The P-loop of *Lm*TK-AP_5_dT is shown as a ribbon. Amino acid residues and ligands are shown as cylinders coloured by atom type with C and P atoms coloured by model. Hydrogen bonds are shown as dashed lines.

The structure of the *Lm*TK-dThd complex (co-crystallised with dThd, AppNHp and magnesium) contained clear electron density for dThd, but none for AppNHp or magnesium, in both molecules in the asymmetric unit. However, there is in addition a phosphate ion bound very close to where the β-phosphate of the AppNHp would be expected from comparison with the structure of *Tm*TK in complex with dThd and AppNHp (PDB ID: 2qq0). As described in detail for *h*TK1 [51], the thymidine molecule is deeply buried between the two domains and is coordinated by hydrogen bonds to the main chain (Fig 3A) while stacking and hydrophobic interactions of side chains hold the thymidine ring in place. In *Lm*TK-dThd, the active site residue E85, essential for catalysis, forms a hydrogen bond to the 5’-oxygen of the ribose ring, well placed to act as the catalytic base, abstracting a proton from the oxygen, which is then enabled to perform a nucleophilic attack on the γ-phosphate of the phosphate donor. The absence of density for AppNHp, compared to the structures of *Ca*TK and *Tm*TK, is a result of the site being sterically blocked in *Lm*TK by residues of the α1-helix of the symmetry-related dimer within the tetramer.

The *Lm*TK–dTTP crystal has a well-resolved dTTP in the thymidine-binding site, the magnesium being coordinated by the phosphates, in all eight protomers in the asymmetric unit. The residues around the dTTP have essentially the same conformation as in the dThd complex, with the exception of E85 that is turned away from the 5’-oxygen of the ribose ring, no longer forming a hydrogen bond. As for the *h*TK1-dTTP structure (PDB ID: 1xbt), the γ-phosphate of the dTTP lies in the position expected for the ATP β-phosphate, explaining why this ligand acts as an inhibitor.

In the *Lm*TK-AP_5_dT complex there are two chains in the AU, both with well-defined density for the dThd moiety and the five phosphates. The coordination of dThd is essentially identical to that in the *Lm*TK-dTTP complex, with E85 in the inactive conformation. Only chain A exhibits good density for the adenosine, which is much less well ordered in Chain B. However, in Chain A, the adenosine is flipped by about 180° from the position expected from the *Ca*TK and *Tm*TK complexes, and lies on the surface of α/β-domain, partially covered by the poorly ordered 42–59 loop. In *Lm*TK-AP_5_dT, the phosphates have different positions from those in the *Lm*TK-dTTP and the *Tm*TK-dThd-AppNHp complexes. Four of the five phosphates of the AP_5_dT show identical positions to those in the *h*TK1-AP_4_dT complex (PDB: 2orv, [39]: the adenine was also not visible in the latter structure. The binding site for adenine expected from the *Ca*TK and *Tm*TK structures is again blocked by the adjacent dimer, just as in the *Lm*TK-dThd complex. Thus while the residues forming the thymidine-binding site have identical conformations in all three *Lm*TK complexes, it is not possible to identify the adenine coordinating residues from the present structures.

Comparison of the active sites of *Lm*TK and the human enzyme does not reveal major differences, which is reflected in the high similarity of kinetic parameters for dUrd, 5F-2’dU and AZT (Table 2).

### Quaternary structure

All type II TK structures in the PDB are tetramers, and analysis using PISA [50] suggested the tetramer as the likely biological entity in all the crystal forms of *Lm*TK. The *Lm*TK-dThd and *Lm*TK-AP_5_dT crystals contain two protomers (Fig 2C) per asymmetric unit, with the tetramer sitting on a crystallographic 2-fold axis (Fig 2D), while the *Lm*TK-dTTP asymmetric unit contains two tetramers. That the tetramer is the stable oligomeric state in solution is confirmed by the AUC analysis below. As discussed extensively for other TK structures, e.g. *Tm*TK [39], there are two protomer-protomer interfaces, the first defined by the central β-sheets of two protomers, which come together in an anti-parallel orientation to form a continuous 12-stranded β-sheet. The second, between dimers in the tetramer, centres on the anti-parallel interaction of the long α1-helices of the α/β-domains of two adjacent subunits.

In the absence of phosphate donor, TK structures form a closed tetramer with the long α1-helices of the α/β-domains interacting directly. While the protomer conformation does not change upon phosphate donor binding, in the presence of the phosphate donor the tetramers are more open with the α1-helices moving ~ 3 Å apart leaving space for the nucleobase. *Lm*TK forms closed tetramers in all three complexes, which explains the lack of density for the adenine in *Lm*TK-dThd and its unexpected position in *Lm*TK-AP_5_dT.

### Oligomeric state in solution

**Fig 4:**
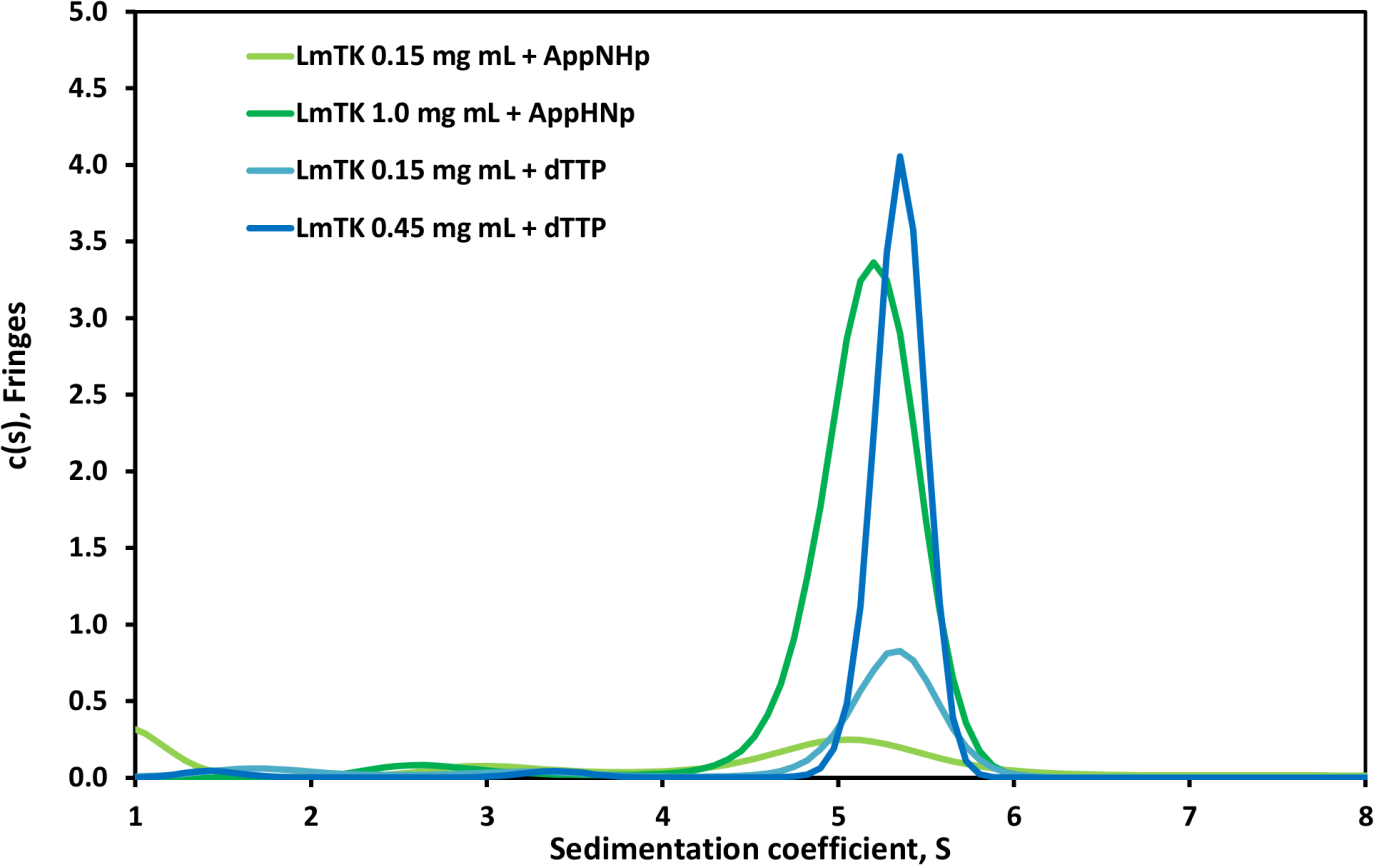
Analytical ultracentrifugation of C-terminally His-tagged *Lm*TK. Superposition of the c(s) distributions of the unliganded *Lm*TK and *Lm*TK-dThd-AppNHp samples are shown at concentrations in the range of 1–8 S.

To confirm the tetramer as physiologically relevant oligomeric state, analytical ultracentrifugation was carried out on unliganded *Lm*TK and enzyme in complex with dThd and AppNHp, each at two different protein concentrations (Fig 4). To obtain unliganded protein, dTTP-containing *Lm*TK was dialysed extensively against ligand-free buffer, while the dialysis buffer used for the *Lm*TK-substrate complex contained the ligands.

At high protein concentrations, the sedimentation coefficients were 5.1 and 5.4 S for the dThd-AppNHp complex and unliganded *Lm*TK, respectively. SEDFIT analysis using standard c(s) distribution model showed frictional ratios of approximately 1.3 resulting in a molecular weight estimate of ~90 kDa, in agreement the value of 84.7 kDa expected for a tetramer, within experimental error. The samples at lower protein concentration showed very similar sedimentation coefficients rendering a concentration-dependent change of oligomeric state unlikely.

### Conclusions

While the full-length enzyme could be expressed in sufficient yield in *E*. *coli* for the kinetic studies, only the C-terminally truncated version of *Lm*TK, spanning amino acids 2–185, expressed soluble in sufficient amounts for structural studies, and required expression in baculovirus to provide stable protein in useful yield. Thus structural work was limited to the core protein with high homology to other TK structures in the PDB. However, analysis and comparison of the *Lm*TK structures with *h*TK1 alongside kinetic data, allow some conclusions about the potential use of TKs as potential targets for anti-leishmanial chemotherapeutics.

The *Lm*TK structures form tightly packed tetramers with a blocked phosphate donor site and are closely similar to those of other species (*h*TK1, [36,51]; *Uu*TK, [52]; *Ba*TK and *Bc*TK, [53]; *Tm*TK, [38,39]; *Ca*TK, PDB Code 1xx6). Furthermore, the tetramer was confirmed by AUC as the oligomeric state in solution. Analysis of the active site revealed no major differences in the region coordinating the thymidine moiety. While there is a difference in the length of the flexible loop region, missing in the structures of *Lm*TK and *h*TK1, this is a crystallographic artefact. While *h*TK1 has the residues interacting with the ribose ring (D58) and the transition state (R60), these residues are disordered in most of the *Lm*TK structures hinting at a more relaxed binding or might be a crystallographic artefact. However, it is ordered in the structure of *Lm*TK:AP_5_dT with the side chain conformations of D58 and R60 being identical to hTK1.

Kinetic measurements of full-length *Lm*TK showed high substrate specificity for its physiological substrates dThd and dUrd. Despite the differences in substrate selectivity found between the *L*. *major* and *T*. *brucei* TKs, a major role in the phosphorylation of Thd and dThd can be proposed for both enzymes. Moreover, we were able to confirm dTTP and AP_5_dT as potent inhibitors of *Lm*TK. We showed AZT and 5’-modified dUrd to be readily phosphorylated by *LmT*K and this compound was shown previously to inhibit *Leishmania* intracellular amastigote growth and to exhibit potential as an antiprotozoal agent [54]. However, the lack of major structural differences in the active site of *Lm*TK compared to the hTK1, further validated by the similarity in their kinetic parameters for dUrd, 5F-2’dU and AZT (Table 2), might render the design of *Lm*TK-specific ligands challenging. Nevertheless, the data obtained for *Lm*TK provide a basis for the design of selective inhibitors that may have potential for the treatment of leishmaniases.

## Supporting Information

S1 Fig Multiple sequence alignment of type II TKs. The secondary structure elements from *Lm*TK-dThd are displayed with α-helices as spirals and β-strands as arrows. Green bar: P-loop, yellow triangles: zinc-coordinating cysteine residues, red stars: residues coordinating dThd *via* main chain hydrogen bonds, orange stars: residues coordinating dThd *via* stacking or hydrophobic interactions of the side chains. Tc: *Trypanosoma cruzi*, *h*TK1: full-length sequence of human TK, *h*TK1-1W4R: truncated hTK1 used for structure solution (PDB ID 1W4R), Vv: Vaccinia virus, Ba: *Bacillus anthracis*, Uu: *Ureaplasma urealyticum*, Tm: *Thermotoga maritima*. Alignment was carried out using M-coffee [58,59] and Espript 3.0 on http://espript.ibcp.fr [60].
(TIFF)

S2 Fig Disorder prediction of the *Lm*TK sequence. Residue number plotted against probability of disorder, calculated by RONN [32]. The green bar indicates the residue range 2–185 for the constructs used in crystallization experiments.
(TIFF)

S3 Fig Effect of pH on *Lm*TK activity. Experiments were performed at 25 μM dThd and 500 μM ATP. Each point represents the average of three determinations and the error bars represent the standard deviation.
(TIF)
