# Supplementary figures and images for: Structural and Kinetic Characterization of Thymidine Kinase from Leishmania major
Source: PLoS Negl Trop Dis. 2015 May 15;9(5):e0003781. doi: 10.1371/journal.pntd.0003781 (PMC4433323; doi:10.1371/journal.pntd.0003781)

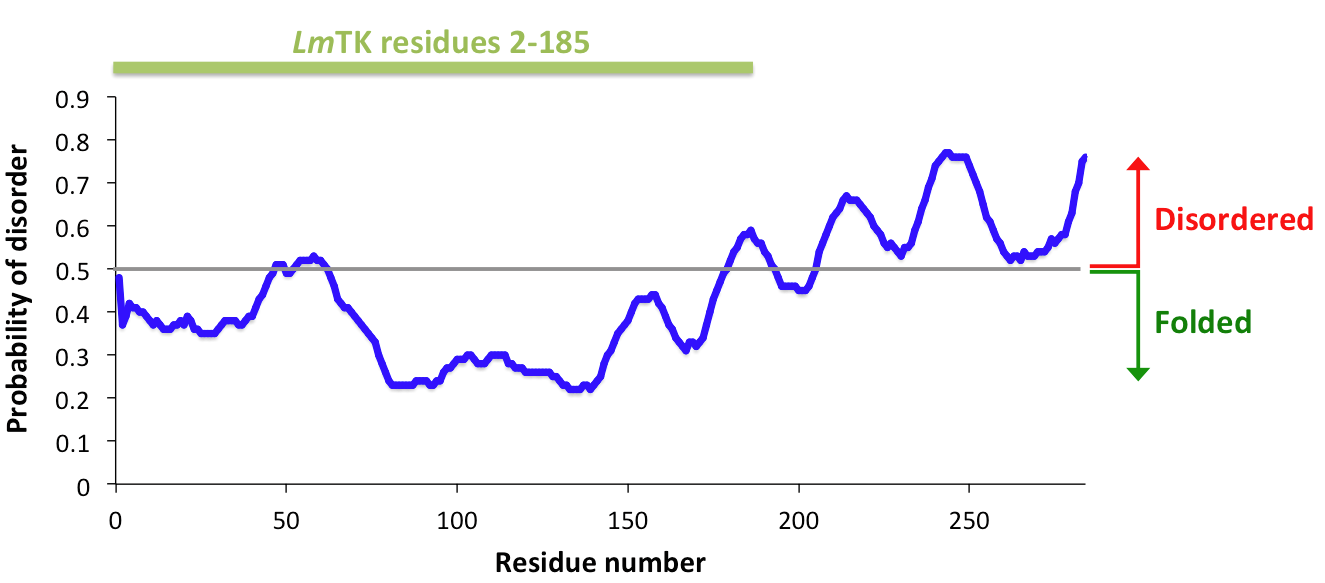

Supplement: S2 Fig — Residue number plotted against probability of disorder, calculated by RONN [32]. The green bar indicates the residue range 2–185 for the constructs used in crystallization experiments. (TIFF) [file pntd.0003781.s002.tiff]
